# Chronic exposure to tramadol induces cardiac inflammation and endothelial dysfunction in mice

**DOI:** 10.1038/s41598-021-98206-2

**Published:** 2021-09-21

**Authors:** Marwa H. Bakr, Eman Radwan, Asmaa S. Shaltout, Alshaimaa A. Farrag, Amany Refaat Mahmoud, Tarek Hamdy Abd-Elhamid, Maha Ali

**Affiliations:** 1 Department of Histology and Cell Biology, Faculty of Medicine, Assiut University, Assiut, 71515 Egypt; 2 Department of Medical Biochemistry, Faculty of Medicine, Assiut University, Assiut, Egypt; 3 Department of Microbiology and Immunology, Faculty of Medicine, Assiut University, Assiut, Egypt; 4 Department of Human Anatomy and Embryology, Faculty of Medicine, Assiut University, Assiut, Egypt; 5 Department of Basic Medical Sciences, Unaizah College of Medicine and Medical Sciences, Qassim University, Unaizah, Kingdom of Saudi Arabia; 6 Department of Anatomy, College of Medicine, Bisha University, Bisha, Kingdom of Saudi Arabia; 7 Department of Biochemistry, Sphinx University, Assiut, Egypt

**Keywords:** Biochemistry, Cell biology, Electron microscopy

## Abstract

Tramadol is an opioid extensively used to treat moderate to severe pain; however, prolonged therapy is associated with several tissues damage. Chronic use of tramadol was linked to increased hospitalizations due to cardiovascular complications. Limited literature has described the effects of tramadol on the cardiovascular system, so we sought to investigate these actions and elucidate the underlying mechanisms. Mice received tramadol hydrochloride (40 mg/kg body weight) orally for 4 successive weeks. Oxidative stress, inflammation, and cardiac toxicity were assessed. In addition, eNOS expression was evaluated. Our results demonstrated marked histopathological alteration in heart and aortic tissues after exposure to tramadol. Tramadol upregulated the expression of oxidative stress and inflammatory markers in mice heart and aorta, whereas downregulated eNOS expression. Tramadol caused cardiac damage shown by the increase in LDH, Troponin I, and CK-MB activities in serum samples. Overall, these results highlight the risks of tramadol on the cardiovascular system.

## Introduction

Tramadol is central acting and widely used opioid analgesic for moderate to severe pain relief^1^. The analgesic effect of tramadol is attributable to its agonistic action on opioid receptors and its inhibitory effect on neuronal reuptake of serotonin and norepinephrine, leading to the blockage of the transmission of nociceptive stimuli in the central nervous system^2,3^.

Clinical studies have described extensive tramadol prescription for managing patients with chronic painful conditions, with the number of prescriptions expected to rise^4–8^. Moreover, tramadol has addiction potential. Abuse is prevalent in areas with high availability, like the Middle East. Most addicts that abuse tramadol takes high doses at once^9^. Unfortunately, multiple adverse effects were developed in patients with prolonged tramadol therapy^10–13^. Additionally, experimental animal studies have associated prolonged tramadol with several organ systems dysfunction and damage, namely cerebral cortex, lung, testis, and liver^14–17^.

Oxidative stress has been implicated in drug intoxication, including prolonged administration of tramadol^16,18^. Excessive production of ROS leads to the peroxidation of membrane lipids, protein, and DNA damage^19^. Additionally, oxidative stress and increased ROS production lead to impairment of mitochondrial respiratory chain, oxidative damage of mitochondrial DNA, and hence mitochondrial dysfunction^20^. Inflammation and increased production of pro-inflammatory cytokines are also manifestations of excess ROS production^21,22^. Interestingly, chronic opioid treatment has been associated with mitochondria-dependent apoptosis and mitochondrial dysfunction^23^.

Several studies have implicated oxidative stress in cardiovascular diseases. In this regard, cardiovascular conditions such as heart failure^24^, endothelial dysfunction^25^, and myocardial ischemia–reperfusion injury^26^ are attributable to oxidative stress-associated mitochondrial dysfunction. Additionally, increased ROS production compromises the function of endothelial nitric oxide synthase enzyme (eNOS) and NO production^27,28^. Defective nitric oxide (NO) production characterizes endothelial dysfunction in several vascular pathological conditions^29^. Moreover, ROS inactivate NO endothelium-dependent vasodilation^30^. These data highlight the involvement of ROS in tissue damage, including heart and blood vessels^31^.

At present, data regarding the effect of opioids on the cardiovascular system are controversial^32,33^. In this regard, the Ogungbe group reported a lack of association between opioid treatment and coronary artery diseases^33^. On the other hand, Solomon and colleagues have reported increased risk for cardiovascular events in arthritis patients taking opioid^32^. Recently, Musich and co-workers demonstrated that 15% of chronic tramadol users had been hospitalized for cardiovascular diseases^34^. Since there is limited literature specifically describing the effects of tramadol on the heart and blood vessels, therefore, it is imperative to characterize the effect of tramadol on the cardiovascular system and the signaling pathways involved.

In the present study, we aimed to characterize the effect of tramadol usage on the mice's heart and aorta. We evaluated the levels of cardiac damage biomarkers and assessed the effect of tramadol on the expression of oxidative stress and inflammatory markers together with the assessment of eNOS and VEGF. Additionally, we performed histopathological examinations of the heart and aortic tissues after exposure to tramadol.

## Materials and methods

### Animals and experimental design

The experimental design was approved by the Institutional Animal Care and Ethics Committee of the Faculty of Medicine, Assiut University. All procedures were performed in accordance with the relevant guidelines and regulations of the Institutional Review Board. This study has been performed in accordance with the ARRIVE guidelines.

Twenty C57BL/6 mice, eight weeks old, were purchased from the Assiut University animal house. They were housed in clean, suitably ventilated cages at 25 ± °C with a 12/12 h light/dark cycle and had free access to water and food. They were acclimatized to their environment for 1 week before starting the experiment.

Mice were divided into two groups (n = 10) each: Sample size was calculated using G*Power 3 software^35^. A calculated minimum sample of 20 mice (divided into 2 equal groups) was needed to detect an effect size of 0.3 in the variance of creatine kinase myocardial band (CK-MB) level and/or lactate dehydrogenase (LDH), with an error probability of 0.05 and 80% power. On expecting 20% attrition, the final sample size will be 20 animals per group (n = 20). Group 1: included mice that received physiological saline solution for 4 successive weeks and serving as control. Group 2: had mice that received tramadol (Tramadol tablets, each contains 225 mg tramadol hydrochloride, Mina-pharm, Egypt) orally by gastric intubation in a dose of 40 mg/kg body weight^36^ dissolved in saline daily for 4 successive weeks.

At the end of the experiment, all mice were sacrificed. Heart and aorta tissue were harvested and divided into two parts. One part was snap-frozen then stored at − 80 °C for qPCR, and the other part was prepared as appropriate for histological studies. Blood samples were collected in dry centrifuge tubes. Sera were separated off and frozen at − 20 °C till further use.

### Histological analysis

For light microscopy, specimens from the left ventricular myocardial and thoracic aorta of all mice were obtained; they were fixed in 10% neutral buffer formalin, dehydrated in ascending grades of ethanol, cleared in xylene, infiltrated and embedded in paraffin, and sectioned at 5 μm thickness. Sections were stained with hematoxylin and eosin (H&E) for general histological evaluation or Masson’s trichrome stain for the demonstration of collagen fibers according to previously published methods^37^.

### Immunohistochemistry

Paraffin sections of cardiac muscle and aorta (5 μm) were mounted on positively charged glass slides with poly-l-lysine and stained with TNF-α (P300A, Thermo scientific, USA), eNOS (PA3-031A, Thermo scientific, USA) and VEGF (PA1-21796, Thermo Scientific, USA) according to the manufacturer’s protocol. Briefly, paraffin sections were deparaffinized in xylene, rehydrated with descending grades of ethanol, and boiled in citrate buffer (pH 6.0) for antigen retrieval. Endogenous peroxidases were blocked by incubation with 3% hydrogen peroxide at 37 °C for 10 min. Sections were incubated with primary antibodies overnight at 4 °C. After washing, sections were incubated with donkey polyclonal secondary antibodies conjugated with horseradish peroxidase (1:5000) (Thermo Scientific, USA) and for 1 h at room temperature. The reaction was detected using 0.05% diaminobenzidine. Sections were counterstained with Mayer’s hematoxylin, mounted, and examined under a Leica microscope (Wetzlar, Germany) equipped with a digital camera. We omitted the primary antibodies during the staining of some of our slides for negative control.

### Scanning electron microscopic (SEM) examination

Aortic sections were cut and opened longitudinally to expose the luminal surface. They were rinsed gently with phosphate buffer saline (PBS) to remove surface debris and immersed in a fixative solution of 2% glutaraldehyde and 1% formaldehyde in 0.1 M phosphate buffer pH 7.2, for 2 h. After fixing, tissue sections were dehydrated in a series of alcohols, and liquid carbon dioxide was used to dry the specimens. Dried specimens were mounted on aluminum stubs, fixed in place with colloidal silver, and sputter-coated with gold^38^. A JEOL (J.S.M-5400LV; Japanese Electron Optic Laboratory) was used to view the specimens. Photographs were taken at 15 kV at the Electron Microscopy Unit, Assiut University.

### Transmission electron microscopic (TEM) examination

Small tissue specimens were taken from the heart and aorta, fixed in 2.5% glutaraldehyde in cacodylate buffer (pH 7.4) for 24 h, post-fixed in osmium tetraoxide in phosphate buffer for 2 h. Tissues were rinsed in the same buffer, dehydrated with ascending grades of alcohol, cleared with propylene oxide, and embedded in Epon-812 substitute^39^. Semithin sections (0.5 µm) were cut with a diamond knife, stained with 1% toluidine blue to be observed in the light microscope. Ultrathin sections (80–90 nm) were cut with ultramicrotome, mounted on copper grids, stained with uranyl acetate and lead citrate to be examined by transmission E.M. (JEOL 100 CX, Japan) at 80 kV in Assiut University EM unit.

### Morphometric analysis

Morphometric data were acquired from images of random fields and analyzed by blind histopathologists using ImageJ version 1.52 k (http://imagej.nih.gov/ij/). The thickness of the tunica intima, tunica media, and myocardial fiber diameter was assessed as previously reported^40,41^. Additionally, mitochondrial aspect ratio (AR), an indicator for mitochondrial fragmentation, was calculated as the ratio of the major axis/minor axis of the mitochondria. Further, the number of TNF-α, eNOS, and VEGF positive cells were counted.

### Real-time quantitative PCR

Heart and aorta tissue from mice were homogenized using a rotary homogenizer under the ice. Total RNA was extracted using Trizol reagent (Invitrogen, USA) according to the manufacturer’s protocol. cDNA was synthesized using a high-capacity reverse transcription kit (catalog no. 4368814, Applied biosystems, USA). Quantitative polymerase chain reaction (qPCR) was performed in a StepOnePlus Real-Time PCR system (Applied Biosystems, USA) using Maxima SYBR Green qPCR Master Mix (catalog no. K0251, Thermo Fischer Scientific, USA). GAPDH was utilized to normalize expression data. Results were expressed as fold change by the 2^–ΔΔCT^ method. The primers used are shown in Table 1. A two-step reaction protocol was used with an initial denaturation of 1 min at 95 °C, followed by 40 cycles of 95 °C for 15 s, then 60 °C for 1 min.

### Assay of biochemical markers of cardiac injury

Serum lactate dehydrogenase (LDH) was measured using Mouse Lactate Dehydrogenase (LDH) ELISA Kit (Catalog No: SG-30229, Sinogene, China). Serum creatine kinase-myocardial band (CK-MB) was measured using Mouse Creatine Kinase MB isoenzyme (CK-MB) ELISA Kit (Catalog No: SG-30111, Sinogene, China). Serum Troponin I was measured using Mouse Troponin I (Tn-I) ELISA Kit (Catalog No: SG-30351, Sinogene, China). All kits were used according to the manufacturer's protocol.

### Assay of lipid peroxidation (MDA assay)

Malondialdehyde (MDA) levels in cardiac and aortic tissues were measured using the Lipid Peroxidation MDA Assay Kit (catalog no. MD2529, Biodiagnostic, Egypt) according to the manufacturer’s protocol.

### Statistical analysis

Statistical analysis was performed by GraphPad Prism 8.2.1 Software (https://www.graphpad.com/scientific-software/prism/). The results were expressed as the mean ± S.E.M. Comparisons between groups were performed using a two-tailed Student *t* test. *p* < 0.05 was considered statistically significant.

### Ethics approval

The experimental design was approved by the Institutional Animal Care and Ethics Committee of the Faculty of Medicine, Assiut University. All procedures were performed in accordance with the relevant guidelines and regulations of the Institutional Review Board. This study has been performed in accordance with the ARRIVE guidelines.

### Consent for publication

Participants have consented to the submission of the data.

## Results

### Effect of tramadol on the expression of heart injury biomarkers

**Figure 1:**
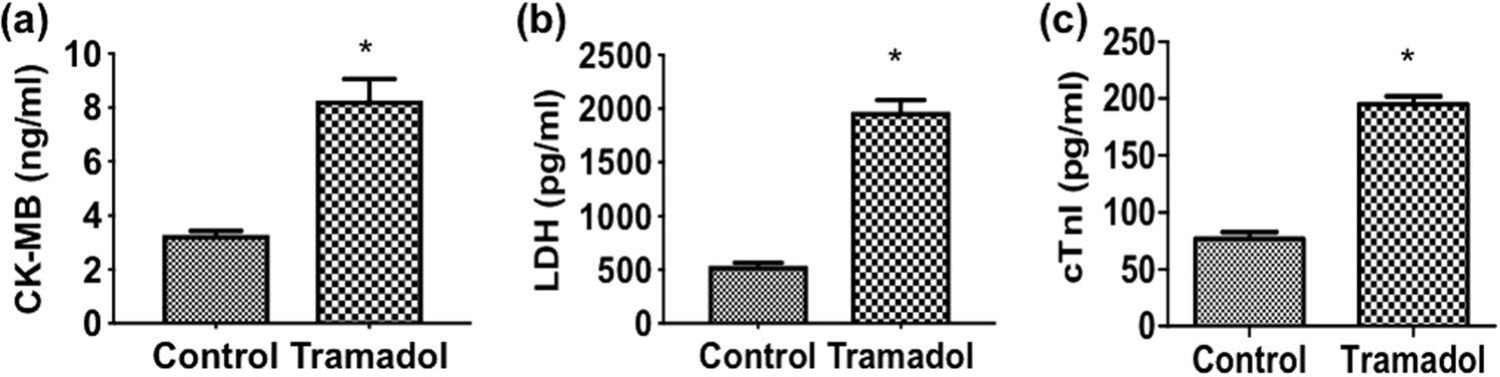
Serum levels of (**a**) CK-MB, (**b**) LDH, and (**c**) cTn-I in each group, n = 10. Data are expressed as means ± SEM, *p < 0.05 for control versus tramadol.

To explore the adverse effect of chronic tramadol treatment, we first assessed the serum levels of heart injury markers: CK-MB, LDH, and Troponin I (cTn-I). Mice treated with tramadol had significantly elevated serum activity of CK-MB, LDH, and cTn-I compared to control animals (*p* < 0.05) (Fig. 1).

### Histological analysis

**Figure 2:**
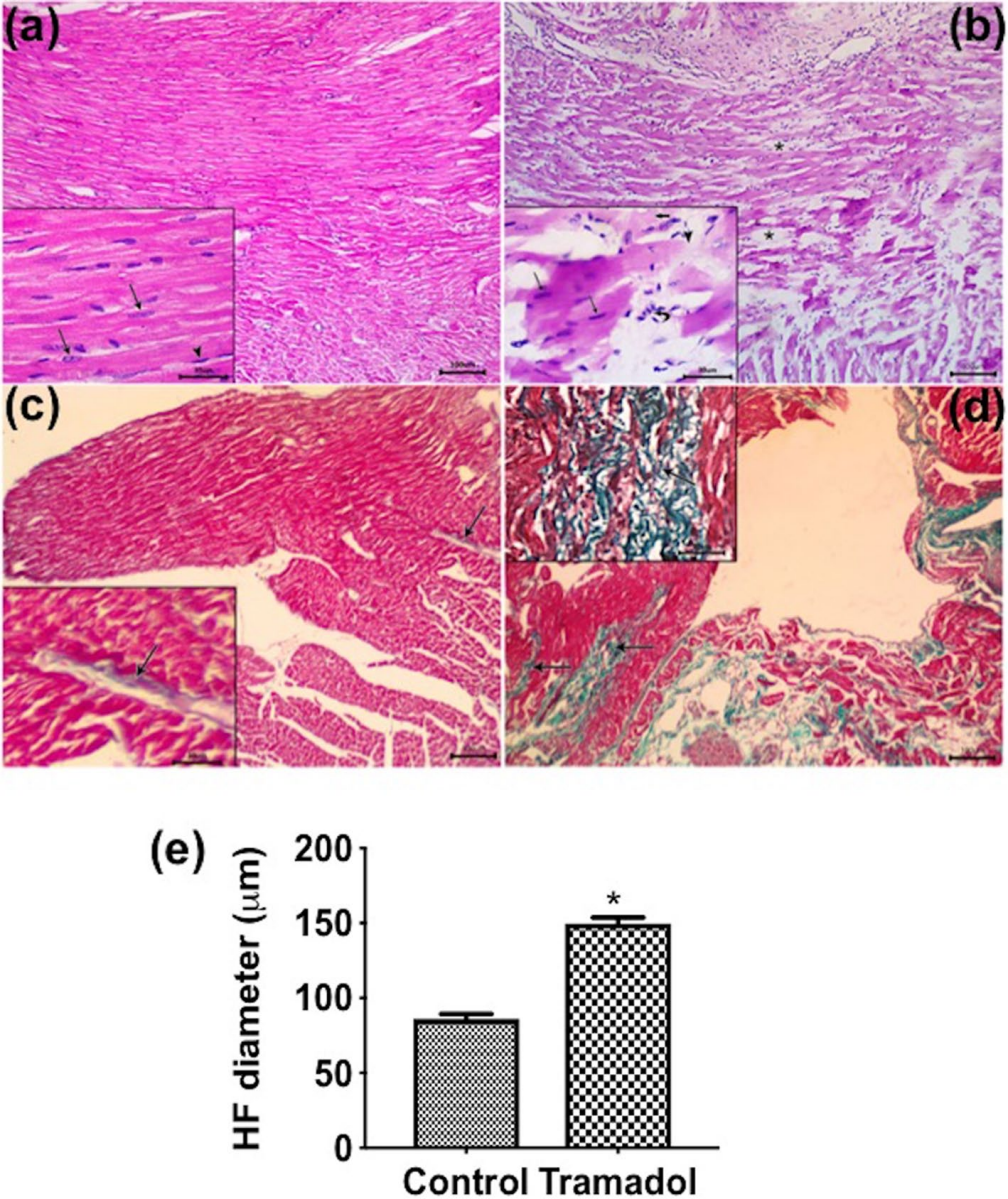
Photomicrographs of a longitudinal section of cardiac muscles stained with hematoxylin and eosin (×100; insets ×400). (**a**) Control group showing branching and anatomizing muscle fibers with central oval vesicular nuclei (arrows in inset) and acidophilic sarcoplasm. The flat nuclei of fibroblasts (arrowhead in inset) are also seen in the interstitial spaces between muscle fibers. (**b**) Tramadol treated group showing separation of cardiac fibers with increased interstitial spaces between them (asterisk). Discontinuity (arrowhead in inset), degeneration (thick arrow in inset), and darkly stained nuclei (arrows in inset) are evident in some muscle fibers. Mononuclear cell infiltration (curved arrow in inset) is also seen between affected widely spaced cardiac muscle fibers. Photomicrographs of a longitudinal section of cardiac muscles stained with Masson's trichrome (×100; insets ×400). (**c**) Control group showing scanty collagen fibers in the interstitial space between muscle fibers (arrows). (**d**) Tramadol treated group showing a marked increase in collagen fibers in the interstitial space between muscle fibers (arrows). (**e**) Heart fiber diameter in control and tramadol mice. n = 10. Data are expressed as the means ± SEM, *p < 0.05 for control versus tramadol.

We then explored the effect of tramadol on the morphology of the heart and aorta. Microscopic examination of H&E-stained sections in the left ventricular myocardium of control mice revealed branching and anatomizing muscle fibers with central oval vesicular nuclei and acidophilic sarcoplasm. These muscle fibers were separated by narrow interstitial spaces containing fibroblasts and small blood capillaries (Fig. 2a). In contrast, left ventricular myocardial sections of mice treated with tramadol showed separation of cardiac fibers with increased interstitial spaces between them; some cardiac muscle fibers appeared pale stained while others had deeply stained sarcoplasm. Additionally, discontinuity, degeneration, and darkly stained nuclei were evident in some muscle fibers. Mononuclear cell infiltration was also seen in between affected widely spaced cardiac muscle fibers (Fig. 2b).

To confirm our observation, we measured the left ventricular cardiac muscle fibers diameter. As expected, treating mice with tramadol significantly decreased the diameter of cardiac muscle compared to control animals (*p* < 0.05) (Fig. 2e).

To better demonstrate collagen fibers, we stained our heart sections with Masson's trichrome stain. Control mice showed scanty collagen fibers in the interstitial spaces between muscle fibers (Fig. 2c). On the other hand, we observed a marked increase in collagen fibers in the interstitial spaces between muscle fibers of tramadol-treated mice (Fig. 2d).

**Figure 3:**
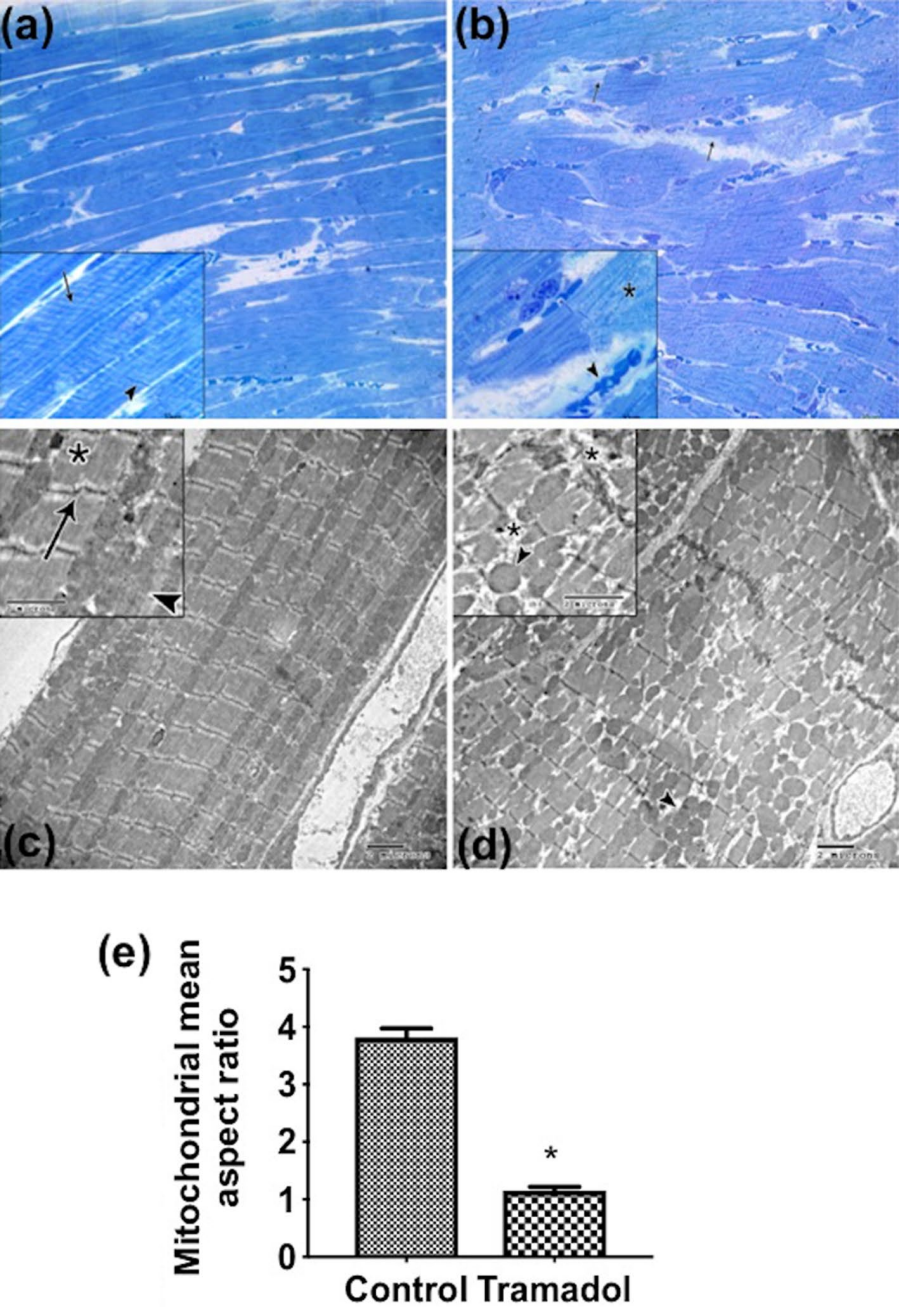
Semithin sections of longitudinally cardiac muscle fibers stained with toluidine blue (×400; insets ×1000). (**a**) Control group showing branching and anatomizing muscle fibers with cross striation along the length of muscle fibers (arrow in inset). Intercalated disks also appear as dark lines (arrowhead in inset). (**b**) tramadol treated group showing disruption in muscle fibers (arrows) and pale stain areas with the disappearance of the intercalated disc (asterisk in inset). Congested blood vessels are also detected (arrowhead in inset). Transmission electron micrographs of cardiac muscle fibers (×3600; insets ×4800) (**c**) The sarcoplasm of control cardiomyocytes composed of longitudinal arrays of cylindrical myofibrils. Each myofibril has myofilaments arranged in sarcomeres (asterisk in inset) that extended between two Z lines (arrows in inset). Elongated mitochondria are regularly arranged in rows between the myofibrils (arrowheads in inset) and often mitochondrion extent the full length of a sarcomere. (**d**) cardiomyocytes from tramadol treated group have areas with disorganization of sarcomeres, fragmentation of myofilaments (asterisk in inset), and accumulated groups of circular rather than elongated mitochondria are seen (arrowheads). (**e**) Aspect ratio. n = 10. Values represent mean ± SEM. *p < 0.05 for control versus tramadol.

On semithin sections stained with toluidine blue, the control group revealed cross striation along the length of muscle fibers, and intercalated disks also appeared as dark lines which extend transversely across the muscle fiber in an irregular manner (Fig. 3a). However, in tramadol treated group, disruption in muscle fibers, degenerative changes with pale stained areas, and congested blood vessels were detected (Fig. 3b).

To further explore the effect of tramadol on cardiac muscles, we processed our heart samples for transmission electron microscope (TEM). The sarcoplasms of control cardiomyocytes were composed of longitudinal arrays of cylindrical myofibrils. Each myofibril had myofilaments arranged in sarcomeres that extended between two Z lines (arrows). Sarcomeres were composed of dark bands (A) and two hemi light bands (I). Additionally, we observed elongated mitochondria regularly arranged in rows between the myofibrils and often mitochondrion extent the entire length of a sarcomere (Fig. 3c). In contrast, cardiomyocytes from tramadol-treated mice showed areas with disorganization of sarcomeres, fragmentation of myofilaments, and accumulated groups of circular rather than elongated mitochondria (Fig. 3d).

We measured mitochondrial aspect ratio (AR; major axis/minor axis) indicative of mitochondrial fragmentation. There was a significant decrease in AR of mitochondria of the tramadol treated group compared to the control group (Fig. 3e), indicating that the mitochondria tended to be more fragmented.

**Figure 4:**
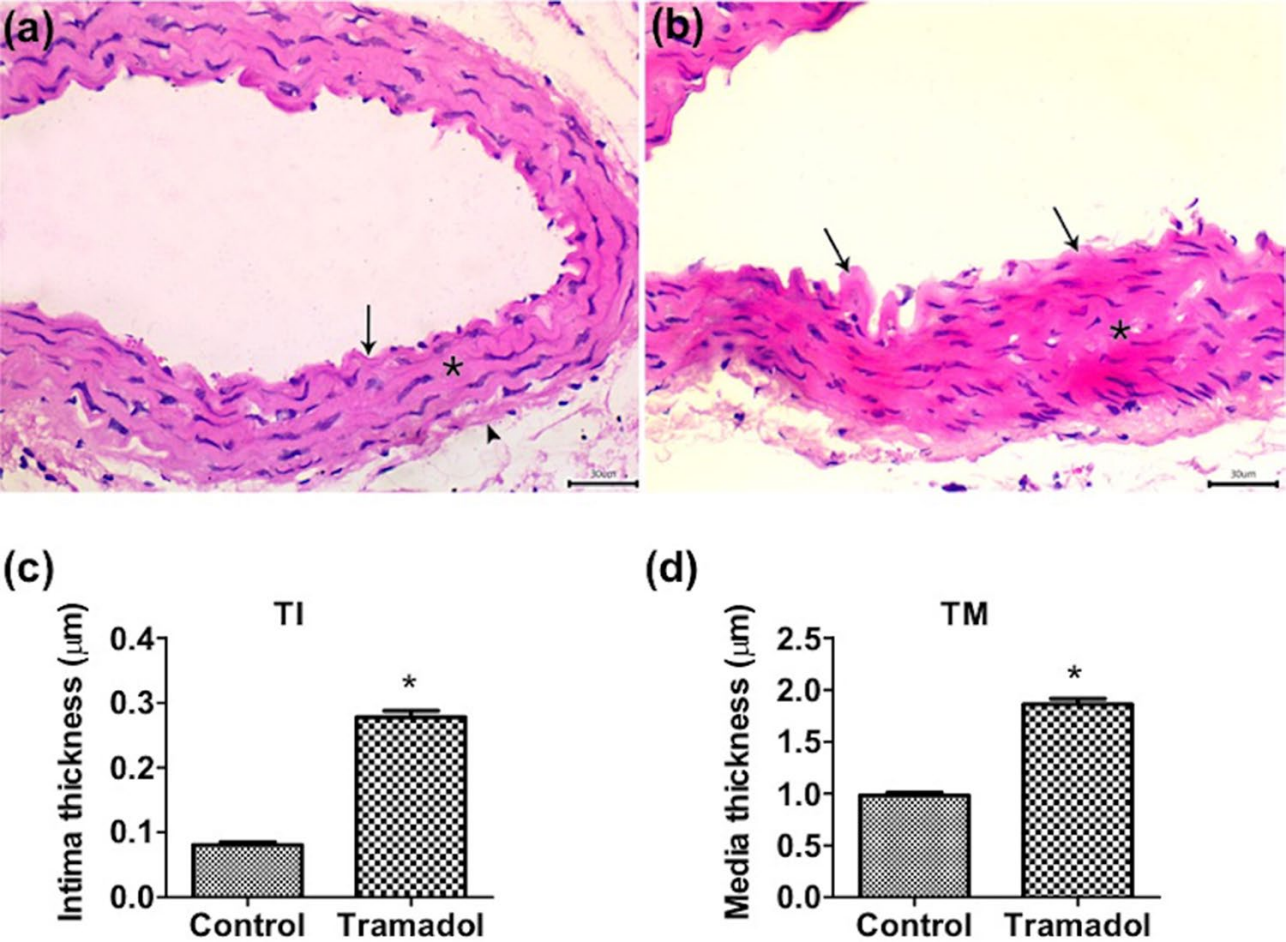
Photomicrographs in a section of thoracic aorta stained with hematoxylin and eosin stains (×400). (**a**) Normal morphology of aortic wall of the control group with tunica intima (arrow), media (asterisk), and tunica adventitia (arrowhead) are seen. (**b**) Tramadol treated group showing thickening of tunica intima (arrows). The irregular arrangement of smooth muscle fibers and elastin lamella of tunica media is seen (asterisk). (**c**) Graphical representation of thickness of the tunica intima (TI) of the thoracic aorta of control and tramadol mice. (**d**) Graphical representation of the thickness of the tunica media (TM) of the thoracic aorta of control and tramadol treated mice. n = 10. Values represent mean ± SEM. *p < 0.05 for control versus tramadol.

To investigate the effect of tramadol on the aorta, we first stained sections from the thoracic aorta with H&E. Regular tunica intima (TI), media (TM), and adventitia layers of the thoracic aorta were seen in sections from control mice (Fig. 4a). On the other hand, aortic sections from mice treated with tramadol revealed thickening of tunica intima and irregular arrangement of smooth muscle fibers and elastin lamella of tunica media (Fig. 4b). The thickness of the intima and media was measured in both control and tramadol-treated groups. Statistical analysis of the measured aortal wall (intima and media) thickness showed a significant difference between both groups (*p* < 0.05) (Fig. 4c,d).

**Figure 5:**
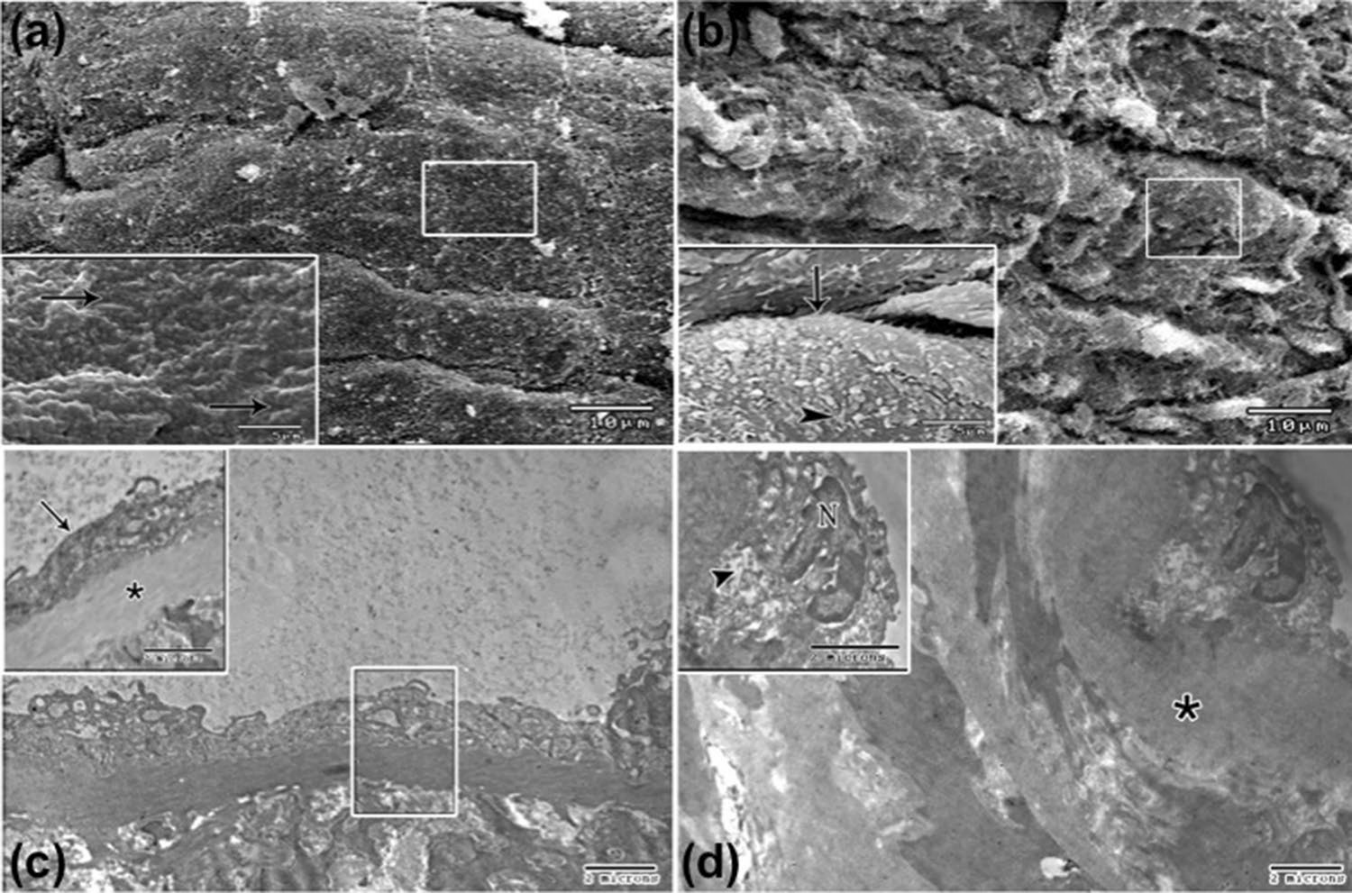
Scanning electron micrograph of mice aortic endothelium (×2000; insets ×5000). (**a**) Control group is showing relatively flat endothelial cells (arrows in inset). (**b**) Tramadol treated group showing raised endothelial cells (arrows in inset) with microvillus-like projections are apparent projecting from the luminal membranes (arrowheads in inset). Notice: white square indicates the area of magnification. Transmission electron micrographs of mice aortic endothelium (×4800; insets ×7200) showing (**c**) the luminal surface of a control group consisting of a single layer of endothelial cells (arrow in inset) in alignment with the internal elastic lamina (asterisk in inset). Notice: white square indicates the area of magnification. (**d**) Irregular alinement of endothelial cells with irregular internal elastic lamina (asterisk) are seen in tramadol treated group. The endothelial cell has an irregular shape nucleus (N) and rarified cytoplasm (arrowhead in inset).

Then we employed a scanning electron microscope to examine the adluminal surface of endothelial cells. Sections from control mice showed aortic endothelium with relatively flattened endothelial cells. The cell outlines were easily resolved (Fig. 5a). The luminal surface of the aorta from the tramadol treated group revealed raised endothelial cells with microvillus-like projections projecting from the adluminal membranes (Fig. 5b).

We also processed our aortic sections for TEM. The tunica intima of the aorta of control mice showed a single layer of endothelial cells aligned with the internal elastic lamina (Fig. 5c). In contrast, the tramadol-treated group showed irregular alignment of endothelial cells with irregular internal elastic lamina. Endothelial cells had an irregular-shaped nucleus and rarified cytoplasm (Fig. 5d).

### Effect of tramadol on the expression of inflammatory cytokines

**Figure 6:**
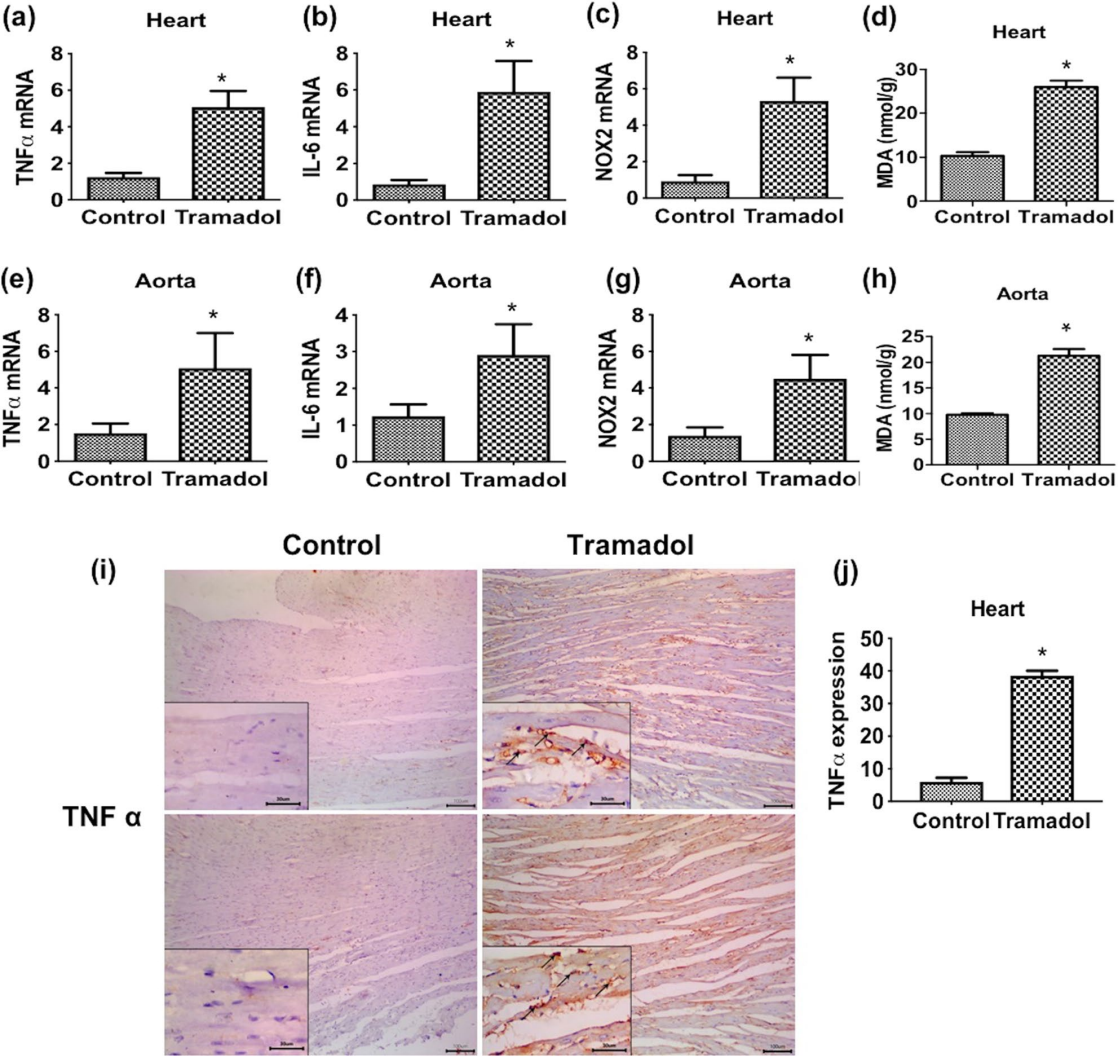
(**a**)–(**c**) Relative quantitative expression of mRNA levels of TNF α, IL6, and NOX2 in cardiac muscle of control and tramadol treated mice. (**e**)–(**g**) Relative quantitative expression of mRNA levels of TNF α, IL6, and NOX2 in the thoracic aorta of control and tramadol treated mice. All gene expression levels were normalized to GAPDH, n = 10. Data are expressed as means ± SEM. *p < 0.05 for control versus tramadol. (**d**) and (**h**) MDA levels in cardiac muscle and the thoracic aorta of control and tramadol treated mice, n = 10. Data are expressed as means ± SEM. *p < 0.05 for control versus tramadol. (**i**) Immunohistochemistry staining images ×100 (inset: ×400) showing expression of TNF α in cardiac muscle of control and tramadol treated mice. Arrows show the specific staining for the antibody. (**j**) Relative quantification of immunohistochemistry staining for TNF α in cardiac muscle of both groups, n = 10. Data are expressed as means ± SEM, *p < 0.05 for tramadol treated versus control.

We first assessed the effect of tramadol on the relative expression of TNF-α mRNA levels. As shown in Fig. 6a, tramadol significantly increased the relative expression of TNF-α mRNA levels compared to the control group (*p* < 0.05). In the aorta, tramadol also significantly increased the relative expression of TNF-α mRNA compared to control mice (*p* < 0.05) (Fig. 6e). We also investigated the expression of TNF-α using the immunohistochemical technique. We observed weak TNF-α immunoreactivity in the myocardium of control mice. On the contrary, tramadol administration markedly increased TNF-α signals in mice myocardium (Fig. 6i). To confirm our observation, we counted the number of TNF-α positive cells using ImageJ software. In this regard, tramadol significantly increased the number of TNF-α positive cardiomyocytes compared to untreated animals (Fig. 6j).

We next assessed the relative expression of IL-6 mRNA in the heart and aorta in response to tramadol treatment. In the heart, tramadol significantly increased IL-6 mRNA levels compared to control animals (*p* < 0.05) (Fig. 6b). Similarly, we observed a significant elevation of the relative expression of IL-6 mRNA in the aorta of tramadol-treated mice compared to untreated animals (*p* < 0.05) (Fig. 6f).

### Effect of tramadol on oxidative stress

To identify the mechanisms underlying tramadol-induced cardiac and aortic injury, we assessed the effect of tramadol on the level of NOX2 transcripts in the heart and aorta. In the heart, treatment of mice daily with tramadol at a dose of 40 mg/kg for 4 weeks significantly increased the relative expression of NOX2 mRNA compared to control animals (*p* < 0.05) (Fig. 6c). Similarly, tramadol administration significantly elevated the mRNA levels of the NOX2 enzyme in the aorta compared to the control group (*p* < 0.05) (Fig. 6g). In addition, we measured lipid peroxidation in cardiac and aorta tissues through an assay of MDA levels. Results showed a significant increase in MDA levels in the heart and aorta of tramadol-treated mice compared to the control group (*p* < 0.05) (Fig. 6d,h).

### Effect of tramadol on the endothelial function

**Figure 7:**
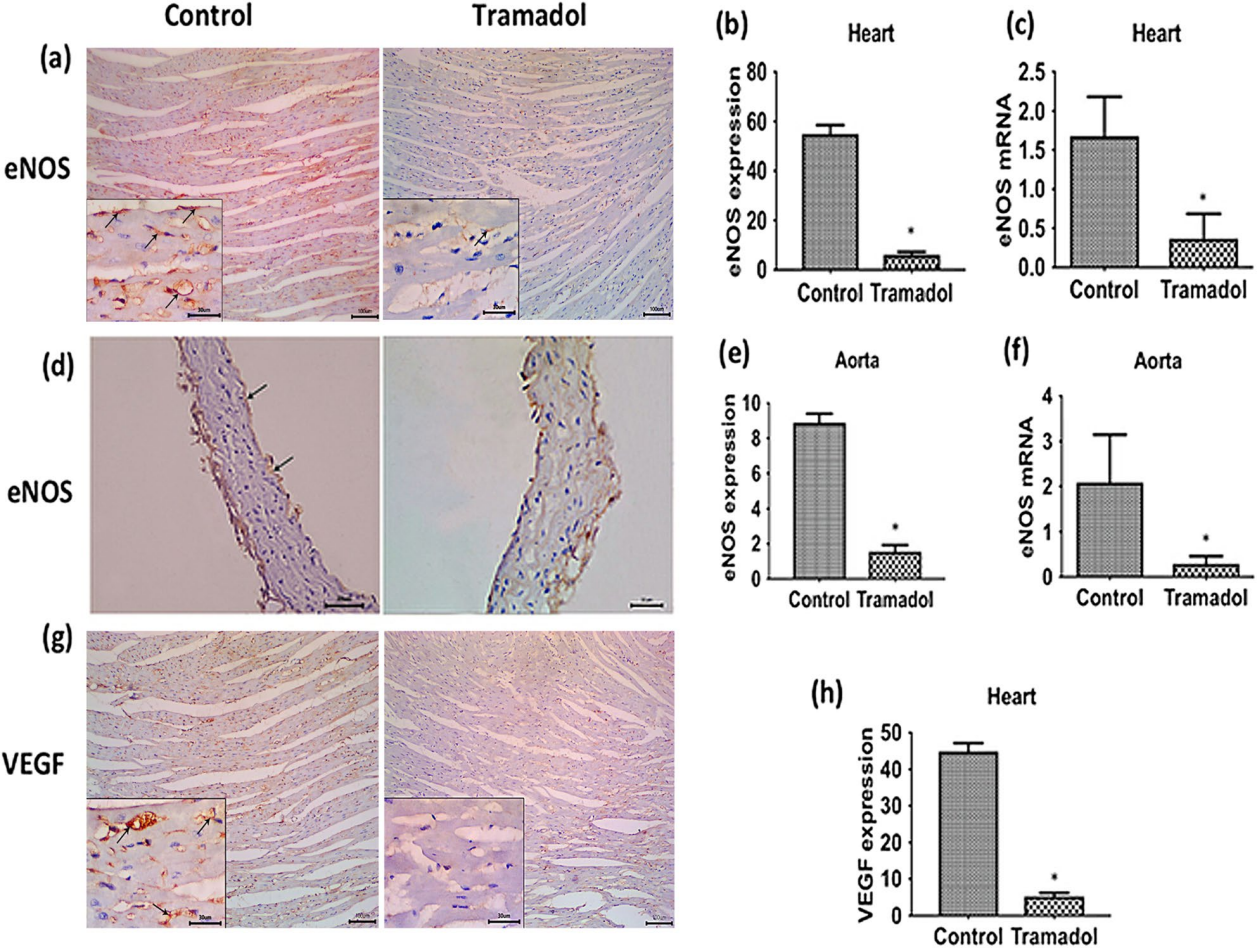
(**a**), (**d**) Immunohistochemistry staining images showing expression of eNOS in cardiac muscle ×100 (inset: ×400) and thoracic aorta ×400 respectively of control and tramadol treated mice. Arrows show the specific staining for the antibody. (**b**), (**e**) Relative quantification of immunohistochemistry staining for eNOS in cardiac muscle and thoracic aorta, respectively of both groups, n = 10. Data are expressed as means ± SEM, *p < 0.05 for control versus tramadol. (**c**), (**f**) Relative quantitative expression of mRNA levels of eNOS in cardiac muscle and thoracic aorta respectively of control and tramadol treated mice. All gene expression levels were normalized to GAPDH, n = 10. Data are expressed as means ± SEM, *p < 0.05 for control versus tramadol. (**g**) Immunohistochemistry staining images ×100 (inset: ×400) showing VEGF expression in cardiac muscle of control and tramadol treated mice. Arrows show the specific staining for the antibody. (**h**) Relative quantification of immunohistochemistry staining for VEGF in cardiac muscle of control and tramadol treated mice, n = 10. Data are expressed as means ± SEM, *p < 0.05 for control versus tramadol.

To further identify the mechanism of tramadol-induced cardiac and aortic injury, we assessed the expression of endothelial nitric oxide synthase (eNOS) in the heart and aorta. As shown in Fig. 7a, we investigated the expression pattern of eNOS using immunohistochemical techniques in myocardial sections. Sections from control mice showed intense eNOS reaction in capillary endothelial cells of the mice hearts. On the contrary, a marked reduction in the eNOS immunoreactivity was observed in myocardial sections from tramadol-treated mice. To confirm our immunohistochemical data, we counted the number of eNOS positive cells using ImageJ software. We observed a significant reduction in the number of eNOS positive cells in myocardial sections of tramadol-treated mice compared to untreated animals (*p* < 0.05) (Fig. 7b). Tramadol administration also significantly decreased the relative expression of eNOS mRNA compared to untreated animals (*p* < 0.05) (Fig. 7c).

Similarly, we assessed the expression of eNOS in the aorta by immunohistochemistry. We observed intense eNOS immunoreaction in endothelial cells lining the aortae of control mice while marked reduction of eNOS immunoreactivity was seen in aortic sections of tramadol-treated animals (Fig. 7d, arrows) with a significant reduction in the number of eNOS positive cells in aortic sections of tramadol-treated mice compared to untreated animals (*p* < 0.05) (Fig. 7e). Tramadol significantly reduced the mRNA levels of eNOS in the aorta compared to control animals (*p* < 0.05) (Fig. 7f).

Myocardial sections were also immunostained with anti-vascular endothelial growth factor (VEGF) antibodies. As shown in Fig. 7g, we observed intense VEGF reactions in the sarcoplasm of cardiomyocytes of control mice (arrows), while tramadol administration markedly reduced VEGF immunoreactivity. We also counted VEGF-positive cells to confirm our immunohistochemical observations. We found that tramadol administration significantly reduced the number of VEGF-positive cells compared to control animals (Fig. 7h).

## Discussion

Despite its effectiveness in managing moderate to severe pain, tramadol has been associated with several tissues damage^10,12,13^. Additionally, chronic use of tramadol was linked to increased hospitalizations due to CVD^34^. Due to the paucity of data describing its actions on the cardiovascular system, we sought to investigate the effect of chronic tramadol treatment on mice heart and aorta. Here, we showed that tramadol increased the serum levels of cardiac injury markers; creatine kinase-myocardial band (CK-MB) and lactate dehydrogenase (LDH). Additionally, we showed that tramadol induced left ventricular cardiomyocyte degeneration, disorganization of sarcomeres, fragmentation of myofilaments, increased interstitial collagen deposition, and mononuclear cell infiltration. Tramadol also increased tunica intima and tunica media thickness and disorganization of smooth muscle fibers and elastin lamella of the media.

Moreover, tramadol increased heart and aorta NOX2 transcripts. Furthermore, tramadol increased heart and aorta TNF-α and IL-6 expression while decreased the expression of endothelial nitric oxide synthase (eNOS) and vascular endothelial growth factor (VEGF). Our study suggested that tramadol induced heart and aorta injury through induction of oxidative stress and inflammation.

The current study revealed that tramadol administration increased the serum levels of CK-MB, Troponin I, and LDH. These are cardiomyocyte metabolic enzymes, and their serum levels are reliable indicators of myocardial damage^42–44^. This elevation of serum CK-MB and LDH is attributable to their leakage through damaged cardiomyocytes sarcolemma into the circulation as suggested previously^42,45^. Additionally, our histopathological results confirmed the toxic effects of tramadol on the heart and aorta in the form of discontinuity, degeneration of cardiac muscle fibers, thickening of tunica intima, and irregular arrangement of smooth muscle fibers and elastin lamella of tunica media.

Tramadol-induced tissue damage is characterized by oxidative stress that causes cardiomyocyte membrane damage through lipid peroxidation^18,46^. Malondialdehyde (MDA), a secondary lipids peroxidation product, is considered an oxidative stress intensity index^47^. NADPH oxidase 2 enzyme (NOX2) is expressed in endothelial cells and cardiomyocytes^27,48^, and it has been associated with mitochondrial dysfunction and CVD^49,50^. NOX2-induced ROS production is an important contributor to cardiovascular damage^51^.

In the current study, we observed a significant elevation of NOX2 transcripts and MDA levels in the heart and aorta of tramadol-treated mice, suggesting the involvement of transcriptional regulation in tramadol-induced NOX2 expression. Our observation is in agreement with the findings of McLaughlin et al.^51^, who demonstrated that doxorubicin-induced cardiac toxicity was associated with elevated NOX2 expression. In addition, downregulation of NOX2 was associated with reduced production of ROS^43^. In the same line, in vitro experiments in cultured endothelial cells revealed that deletion of NOX2 decreased superoxide production and apoptosis upon starvation^52^. Similarly, several studies have reported the association of high MDA levels with cardiovascular injury^53,54^. Taken together, the cardiac and aortic damage observed with tramadol treatment is probably due to increased oxidative stress-mediated through NOX2 and MDA.

Accumulating data suggest that the production of ROS in excess promotes inflammatory conditions. Inflammatory mediators such as TNF-α and IL-6 released during these conditions are tightly linked to pathological cardiovascular diseases, including drug intoxication^55,56^. Herein, we reported that treating mice with tramadol increased the expression of TNF-α mRNA and/or protein in the heart and aorta. We also showed that the expression of IL-6 mRNA was significantly elevated in animals treated with tramadol. Our data indicate that prolonged treatment of animals with tramadol established inflammation in the heart and aorta.

In agreement with our results, prolonged exposure to tramadol has been reported to increase cerebral expression of TNF-α and IL-6 transcripts^57^. Moreover, Anilkumar et al.^58^ have demonstrated that TNF-α increased ROS production in endothelial cells overexpressing NOX2, suggesting the involvement of NOX2 in TNF-α-induced excess ROS production. Furthermore, treating aortic endothelial cells with IL-6 in vitro increased the expression of NOX2 and ROS production^59^. Similarly, Zhao et al.^60^ showed that NOX2 inhibition decreased ROS, TNF-α, and IL-6 production. Collectively, it is likely that tramadol, through upregulation of NOX2 expression, induced oxidative stress, which triggered the release of pro-inflammatory mediators, TNF-α and IL-6. These mediators further increased ROS production and ultimately lead to heart and aortic abnormalities.

Oxidative stress has been linked to the development of cardiac fibrosis characterized by the alteration of extracellular matrix components (ECM), mainly collagen contents^61^. Cardiac fibrosis increases myocardial stiffness, contributes to cardiac dysfunction, and ultimately leads to heart failure^62^. Herein, we showed increased cardiac collagen deposition in tramadol-treated mice as evidenced by Masson trichrome staining. In line with our study, Tsai et al.^63^ have reported that animals treated with doxorubicin not only showed damaged cardiomyocytes but also developed myocardial fibrosis.

Tramadol-mediated myocardial fibrosis can be explained by several mechanisms. Zhang et al.^64^ showed that damaged cardiomyocytes secrete factors that induce cardiac fibroblasts proliferation, expression of α-smooth muscle actin, migration, and collagen gene expression. Additionally, oxidative damage to myocardial microvascular endothelial cells has been associated with the transformation of endothelial cells to mesenchymal cells (EndMT) and increased production of ECM^63,65^. Interestingly, Huang et al.^66^ reported that mice challenged with lipopolysaccharides showed increased expression of NOX2, elevated ROS levels, and development of cardiac fibrosis; these effects were reversed after NOX2 inhibition. Altogether, tramadol-induced cardiac fibrosis could be attributable to NOX2-induced oxidative stress, which leads to activation of cardiac fibroblasts and/or microvasculature endothelial cells to undergo EndMT and lay down ECM components.

NO is a vasodilator molecule produced by endothelial cells. Dysregulation of NO characterizes endothelial dysfunction, which underlies several vascular abnormalities. This defect is due to the abnormal activity or expression of eNOS, the predominant NOS isoform in endothelial cells^67,68^, or decreased NO bioavailability as a consequence of increased ROS generation^69^. Our results showed that treating animals with tramadol significantly decreased the expression of eNOS in the heart and aorta at both mRNA and protein levels compared to control mice. Moreover, the current SEM and TEM examination support our finding as they revealed endothelium changes. The altered eNOS expression in tramadol-treated animals could be attributable to endothelial cell inflammation. This assumption is further supported by the recent work of Uthman et al.^70^, who demonstrated disturbance of eNOS expression upon treating cultured endothelial cells with TNF-α. In a similar line, Singh et al.^71^ have shown that upregulation of eNOS expression reversed endothelial dysfunction induced by exposure to inflammatory stress. Further, Simplicio et al. (2017) reported that the inhibition of NOX by apocynin reversed the ethanol-induced reduction of eNOS expression and improved the endothelial function of rat-resistant arteries. This could indicate that NOX2-derived ROS and inflammation might have participated in reduced eNOS expression in our model.

In the present study, our immunohistochemical analysis showed that treating mice with tramadol reduced the expression of myocardial vascular endothelial growth factor (VEGF). Mice lacking VEGF were reported to have small heart size and abnormal coronary vasculature^72^. Moreover, direct transfer of the VEGF gene into the myocardium improved cardiac function and promoted angiogenesis in an animal model of myocardial infarction^73^.

Our results showed that tramadol treatment decreased VEGF expression. Previous research described that activation of eNOS is involved in the expression of VEGF^74^. Additionally, Chen and co-workers demonstrated that the eNOS gene delivered directly into the heart reversed cardiomyocytes' apoptosis and increased capillary density in infarcted myocardium^75^. On the other hand, Haddad et al.^76^ reported that treating cultured endothelial cells with oxidized LDL increased ROS, impaired VEGF-mediated cell migration, and endothelial tubal formation. These effects were abolished upon inhibition of NOX with apocynin. These data suggest that NOX2-derived ROS compromised VEGF-mediated angiogenesis.

Interestingly, TNF-α disturbs the capacity of endothelial cells to form capillary tubes in vitro, suggesting the involvement of inflammation in impaired angiogenesis^71^. TNF-α decreases eNOS phosphorylation and hence NO production^28^. Collectively, tramadol impaired VEGF expression could be attributable to the tramadol-induced reduction of eNOS expression and/or NOX2 derived ROS, and inflammation.

## Conclusion

The results of the current study confirmed the toxic effects of tramadol on the heart and aorta. Tramadol treatment induced cardiac damage demonstrated by the increased serum levels of cardiac injury markers and histopathological findings. Moreover, tramadol-induced oxidative stress, inflammation, and endothelial dysfunction. This study provides insights into the mechanisms of tramadol-induced cardiac and aortic injury.

**Table 1:** Primers used in qPCR experiments.

Gene	Accession numder	Sequence(5′ −> 3′)
GAPDH	NM_001289726.1	Forward: 5′-CGTGCCGCCTGGAGAA-3′
		Reverse: 5′-CCCTCAGATGCCTGCTTCAC-3′
eNOS	NM_008713.4	Forward: 5′-TCCGGAAGGCGTTTGATC-3′
		Reverse: 5′-GCCAAATGTGCTGGTCACC-3′
TNF-α	NM_001278601.1	Forward: 5′-GCCTCTTCTCATTCCTGCTTG-3′
		Reverse: 5′-CTGATGAGAGGGAGGCCATT-3′
IL-6	NM_001314054.1	Forward: 5′-ACGGCCTTCCCTACTTCACA-3′
		Reverse: 5′-CATTTCCACGATTTCCCAGA-3′
NOX2	NM_007807	Forward: 5′-ACTCCTTGGGTCAGCACTGG-3′
		Reverse: 5′-GTTCCTGTCCAGTTGTCTTCG-3′

## Data Availability

Data supporting the findings of this study are available from the corresponding author upon reasonable request.

## Abbreviations

CVD: Cardiovascular disease
NOX2: NADPH oxidase 2
eNOS: Endothelial nitric oxide synthase
VEGF: Vascular endothelial growth factor
IL-6: Interleukin-6
LDH: Lactate dehydrogenase
TNF-α: Tumor necrosis factor-alpha
CK-MB: Creatine kinase-myocardial band
